# Commercially Available Eye Drops Containing Trehalose Protect Against Dry Conditions via Autophagy Induction

**DOI:** 10.1089/jop.2020.0119

**Published:** 2021-09-08

**Authors:** Eva Hernandez, Clémence Taisne, Marion Lussignol, Audrey Esclatine, Marc Labetoulle

**Affiliations:** ^1^ CEA, CNRS, Institute for Integrative Biology of the Cell (I2BC), Université Paris-Saclay, Gif-sur-Yvette, France.; ^2^ Service d'Ophtalmologie, Hôpital Bicêtre, APHP, Université Paris Sud, Le Kremlin-Bicêtre, France.; ^3^ Center for Immunology of Viral infections and Autoimmune Diseases (IMVA), IDMIT Infrastructure (http://www.idmitcenter.fr), CEA, Université Paris Sud, Inserm U1184, Fontenay-aux-Roses Cedex, France.

**Keywords:** autophagy, osmoprotection, trehalose, dry eye, artificial tears

## Abstract

***Purpose:*** Evaluation of marketed eye drops with or without trehalose, a nonreducing natural osmoprotector disaccharide, in autophagy modulation and its role in cell survival during desiccation.

***Methods:*** Eye drops containing either sodium hyaluronate (SH) (Hyabak^®^, Thea, France) or a combination of SH with trehalose (Thealose Duo^®^, Thea, France) were compared with control conditions to evaluate the ability to modulate autophagy in human epithelial cells *in vitro*. Autophagy was monitored using LC3, a marker of the autophagic machinery, by fluorescence microscopy and immunoblot analysis. Control and autophagy-deficient cells treated with eye drops were exposed to desiccation to mimic dry eyes and cell survival was evaluated by thiazolyl blue tetrazolium bromide (MTT) assay. Trehalose, a known autophagy inducer was used as a positive control.

***Results:*** Artificial tears containing SH with and without trehalose induce a complete autophagic flux, as indicated by an increase in the number of autophagosomes and autolysosomes, and the accumulation of the lipidated form of LC3 associated with complete autophagy. In addition, there was a synergistic effect of SH for autophagy induction when combined with trehalose, compared with each of the components alone. Survival of cells treated with both eye drops and exposed to desiccation was decreased in autophagy-deficient cells, demonstrating the essential role of autophagy on eye drop protection.

***Conclusions:*** Autophagic flux is induced by SH-containing eye drops, and this phenomenon is enhanced in combination with trehalose. We also demonstrated that autophagy induction is involved in the osmoprotective effects of both trehalose and SH-containing eye drops, to maintain epithelial cell homeostasis in dry conditions.

## Introduction

The prevalence of dry eye disease (DED) is increasing owing to a number of factors including, a greater proportion of elderly in the general population, the pervasiveness of digital devices, and the increased use of heating/air conditioning systems.^1,2^ A study of almost 10 million subjects in the United States reported that the annual prevalence of DED has tripled over the last decade.^3^ Furthermore the incidence and prevalence of DED increased across all age groups.^3^ In China the prevalence of DED is forecast to affect >349 million individuals in the coming years.^4^ A population-based study reported a prevalence of definitive DED in 21.9% of elderly French subjects.^5^

In DED, increased evaporation at the ocular surface leads to hyperosmolarity causing inflammation that results in epithelial and goblet cell loss, which leads to tear film instability that increases hypersomolarity.^6^ If left untreated, this self-perpetuating cycle results in a loss of homeostasis of the tear film, potentially increasing the severity of the disease. However, an innate process called autophagy participates in maintaining homeostasis by mitigating the effects of inflammation and other stressors on corneal cells. Autophagy degrades and recycles cellular compounds allowing the cell to adapt and survive under harsh environmental conditions such as low moisture or dehydration.^7^

Topical lubricants or artificial tears remain the mainstay for dry eye therapy.^8^ Artificial tears act as osmoprotectants, to restore cell volume, reduce cell stress, and inflammation caused by increased osmolarity.^7^ Osmoprotection is a cellular mechanism that protects against osmotic stress. Several osmoprotectors are currently used in artificial tear formulations that decrease the effects of hyperosmolarity, including glycerol, erythritol, taurine, carnithine, and trehalose.^8–10^

Trehalose is a natural nonreducing disaccharide, produced by plants, microorganisms, and insects, that stabilizes protein and membranes.^9^ Trehalose is also known to function as both a bioprotectant and an osmoprotectant.^10^ In ocular tissues, trehalose mitigates the effects of desiccation on corneal cells and delays cellular apoptosis in such conditions.^10^ Although trehalose is known to induce autophagy, little is known about the mechanisms involved and the potential interactions with the other components present in tear substitutes, especially sodium hyaluronate (SH).

Autophagy is an evolutionarily conserved vacuolar cellular process that clears misfolded proteins and damaged organelles.^11^ A double membrane vesicle called autophagosome engulfs cytoplasmic material and fuses with the lysosome to form an autolysosome, where degradation occurs through lysosomal enzymes. Autophagy can be activated during stress and starvation, allowing the cell to protect itself against environmental stress.^11^ Impaired autophagy has been implicated in several pathogenic processes, including neurodegenerative diseases, diabetes, and cancer.^12^ Reduced autophagy has also been associated with accelerated aging.^12^ Similarly, defective autophagy has been associated with inflammation in ocular surface diseases.^13–15^ Defective autophagy associated with lacritin monomer deficiency in dry eye may cause a loss of ocular surface homeostasis, and this was reversed by topical lacritin in a dry eye mouse model.^14^

Autophagy increases cellular stress tolerance thereby increasing the threshold for cellular apoptosis using several mechanisms, including the selective removal of damaged, apoptosis-inducing mitochondria.^16,17^ In a murine model of DED, trehalose protected against apoptosis of corneal and conjunctival cells (including goblet cells)^18,19^ and against UV-induced oxidative damage in leporine corneal cells.^20^ In addition, there was a marked decrease in inflammatory cytokines in the conjunctiva.^19^ Hence, enhancing autophagy may be a reasonable approach to treat ocular surface diseases.

This *in vitro* study evaluates the effect of trehalose alone, and as a compound of marketed eye drops that also contain SH, for the ability to upregulate autophagy in human epithelial cells in culture.

## Methods

### Cell culture

HeLa cells expressing green fluorescent protein-LC3 (GFP-LC3; provided by Aviva Tolkovsky)^21^ or red fluorescent protein-GFP-LC3 (mRFP-GFP-LC3; provided by David Rubinsztein)^22^ were cultured in Dulbecco's modified Eagle's medium (Gibco^®^ DMEM 41965; Thermo Fisher Scientific, Inc., Waltham, MA) supplemented with 10% fetal calf serum (FCS S181B-500; Dutscher, Brumath, France) and 500 μg/mL G418 (10131035; Thermo Fisher Scientific, Inc.) at 37°C under 5% CO_2_. Human foreskin fibroblasts (HFF) provided by Thomas Shenk (Princeton University, Princeton, NJ), and autophagy-deficient stable cell line (HFF ATG4B C74A)^22^ were maintained in DMEM supplemented with 10% FCS.

### Antibodies and drugs

Anti-LC3 antibody was purchased from Clinisciences, Nanterre, France (PM036 MBL). Anti-actin control antibody was acquired from Merck Millipore (Burlington, MA) (MAB1501). Horseradish peroxidase (HRP)-labeled goat anti-mouse or anti-rabbit secondary antibodies were purchased from Jackson Immunoresearch (West Grove, PA) (115-035-003, 111-035-003). Hyabak^®^ and Thealose Duo^®^ (Thealose^®^ in France) eye drops were provided by Laboratories Thea (Clermont-Ferrand, France) and were diluted by half in DMEM and applied to the cells during different times depending on the experiments.

Earle's balanced salt solution (EBSS) was used during 4 h as a positive control to induce autophagy (11540616; Thermo Fisher Scientific, Inc.). Trehalose (T0167; Sigma) was used at concentration of 40 mM and applied to the cells during different times. To block the autophagic flux, chloroquine (CQ) (C6628; Millipore Sigma, Darmstadt, Germany) was used at a concentration of 50 μM in the last 4 h before cell lysis or fixation.

### Fluorescence microscopy

GFP-LC3 HeLa cells or mRFP-GFP-LC3 HeLa cells were seeded on glass coverslips in 24-well plates at a density of 50,000 cells per well. Cells were treated with Hyabak, Thealose Duo or trehalose for 4, 8, or 24 h. Cells were washed once with phosphate-buffered saline (PBS) and then fixed with 4% paraformaldehyde. Nuclei were stained with DAPI (10184322; Thermo Fisher Scientific, Inc.) and coverslips were mounted using Glycergel^®^ (C0563, Dako; Agilent Technologies, Inc., Santa Clara, CA). Images were obtained with Act1 software using Eclipse 80i epifluorescence microscope (Nikon Corp., Tokyo, Japan). All images were acquired with the same microscope settings (aperture and exposure time) to allow direct comparison. The number of LC3 dots per cell was determined using ImageJ software (NIH, Bethesda, MD) as previously described.^23^ Thresholding of images was performed to detect only LC3 vesicles. The value of the threshold was conserved between each analyzed cell. For each condition, at least 25 cells were analyzed in 3 independent experiments.

### Western Blot

Cells were plated in 24-well plates and treated for 24 h with Hyabak, Thealose Duo or trehalose and CQ was either added or not added, 4 h before lysis to study the autophagic flux. Cells were washed with PBS and lysed in 65 mM Tris, pH 6.8, 4% sodium dodecyl sulfate (SDS), 1.5% β-mercaptoethanol, and placed at 100°C for 5 min. Protein lysates were resolved on 12.5% sodium dodecyl sulfate–polyacrylamide gel electrophoresis (SDS-PAGE), then electrotransferred to a polyvinylidene fluoride membrane (Amersham Plc., Buckinghamshire, United Kingdom). Membranes were blocked for 1 h in PBS 0.1% Tween containing 5% bovine serum albumin (04-100-812-C; Euromedex, Strasbourg, France) before antibody staining overnight at 4°C. HRP-labeled secondary antibodies were used followed by chemiluminescence detection according to the manufacturer's instructions (Immobilon; Millipore). Protein levels were quantified using ImageJ software.

### Cytotoxicity assay

GFP-LC3 HeLa, HFF, and autophagy-deficient (HFF ATG4B C74A) cells were seeded in 96-well plate, and treated with Hyabak, Thealose Duo or trehalose. DMEM was used as a “nontreated” control. After indicated exposure times, cytotoxicity was assessed using thiazolyl blue tetrazolium bromide (MTT) (M5655; Sigma) as recommended by the manufacturer. In brief, media was removed and 0.3 mg/mL MTT was added. After 4 h at 37°C, MTT was removed and MTT crystals were resuspended in 200 μL DMSO (D8418; Sigma). Optical density was measured at 550 nm (using 630 nm as a reference wavelength) with the Infinite T50 microplate reader (Tecan Group Ltd, Männedorf, Switzerland) and Magellan software. Results are expressed as percentage using nontreated cells as control (100%).

Three independent experiments were performed in triplicate. Data were expressed as mean ± standard deviation (SD). One-way analysis of variance (ANOVA) was performed using Prism 7.0 (GraphPad software, San Diego, CA). Values of *P* < 0.001 were considered statistically significant.

### Desiccation assay

HFF and HFF ATG4B C74A cells were seeded at a density of 15,000 cells per well in a 96-well plate. Different treatments were applied overnight. The following day, media was removed from the wells and cells without any liquid were placed under the microbiological safety cabinets with flow for 10 min in standardized conditions to mimic desiccation. Media containing 0.3 mg/mL MTT was then added and cells were placed at 37°C, 5% CO_2_ for 4 h. Absorbance was measured at 550 and 630 nm (reference wavelength) using Infinite F50 microplate reader (Tecan) and Magellan software. Cell survival was expressed as the times-fold increase using desiccated nontreated cells as the reference.

Three independent experiments were performed in triplicate and data were expressed as mean ± SD. ANOVA was performed using Prism 7.0 (GraphPad Software). *P* values <0.05 were considered statistically significant.

## Results

### Artificial tears containing SH and trehalose induce autophagosome accumulation in epithelial cells

**FIG. 1.**
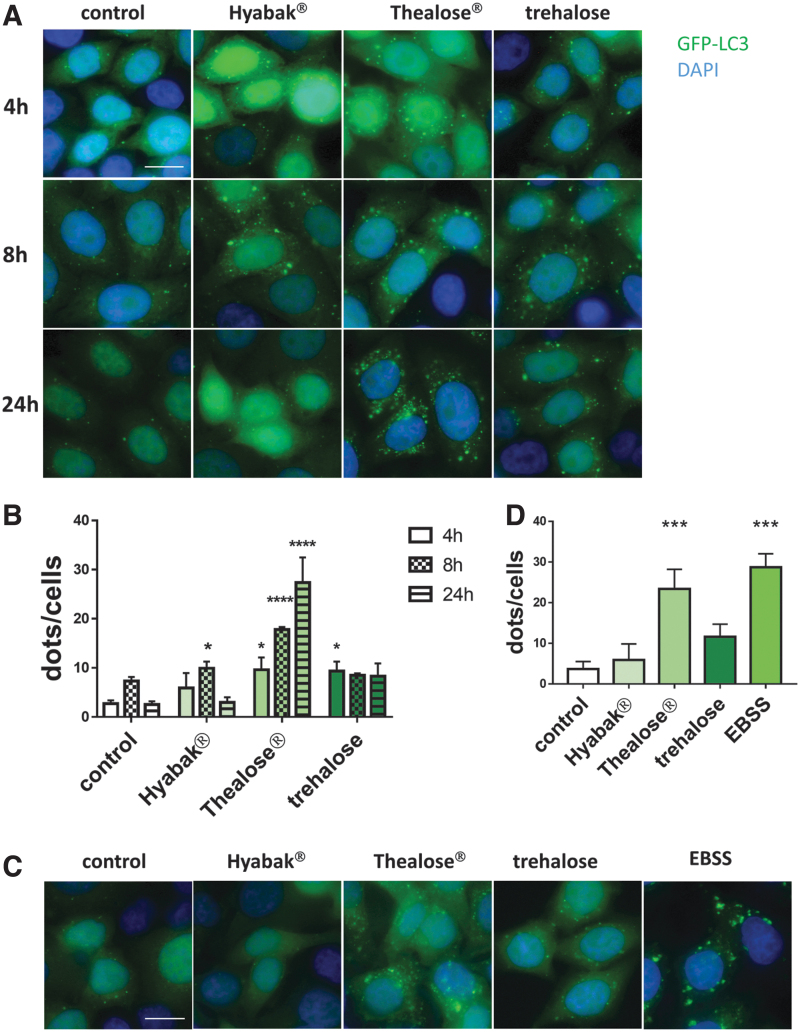
Accumulation of autophagosomes in epithelial cells treated with artificial tears containing sodium hyaluronate and trehalose. **(A, B)** GFP-LC3 HeLa cells were treated with Hyabak or Thealose Duo for 4–24 h. Treatment with trehalose alone at a final concentration of 40 mM was used as a known inducer of autophagy. Nuclei were stained with DAPI. Scale bar = 10 μm **(A)** Representative images. **(B)** Quantification of the number of GFP-LC3 dots per cell. **(C, D)** GFP-LC3 HeLa cells were treated with Hyabak or Thealose Duo during 24 h. Treatment with EBSS (nutrient starvation) was used as a known inducer of autophagy. **(C)** Representative images of 3 independent experiments. Scale bar = 10 μm. **(D)** Quantification of the number of dots per cell. *p < 0.05; ***p < 0.001; ****p < 0.0001 (one-way Anova test) compared with the untreated control cells. EBSS, Earle's balanced salt solution; GFP, green fluorescent protein.

To evaluate the ability of artificial tears containing SH alone (Hyabak) or in combination with trehalose (final concentration 40 mM; Thealose Duo) to modulate the autophagic process, different assays to monitor autophagy were performed in human epithelial cells in culture. The LC3 protein is a classic marker of autophagy because, upon autophagy induction, the lipidated form of LC3 associates with autophagosomal membranes, resulting in the formation of punctuate structures that can be visualized by fluorescence microscopy. Human epithelial cells stably expressing GFP-LC3 were treated with Hyabak or Thealose Duo for 4–24 h. Treatment with trehalose alone at a final concentration of 40 mM in complete medium was used as a known inducer of autophagy. Both Hyabak and trehalose displayed an increased number of GFP-LC3-positive cells with GFP-LC3 dots compared with control cells (Fig. 1A, B). Of interest, treatment with Thealose Duo resulted in an even greater increase in the number of autophagosomes per cell in a time-dependent manner. As amino acid starvation stimulates autophagy for recycling proteins, we compared the autophagy level of cells exposed to artificial tears with cells under deprivation (Fig. 1C, D). Cells were starved in nutrient-free EBSS for 4 h as a positive control and as expected, EBSS treatment increased the number of autophagosomes in HeLa cells.^22^ We observed that a 24-h treatment of a combination of SH with trehalose induced autophagosome accumulation similar to EBSS, whereas artificial tears containing SH moderately increased the number of autophagosomes under the same conditions.

**FIG. 2.**
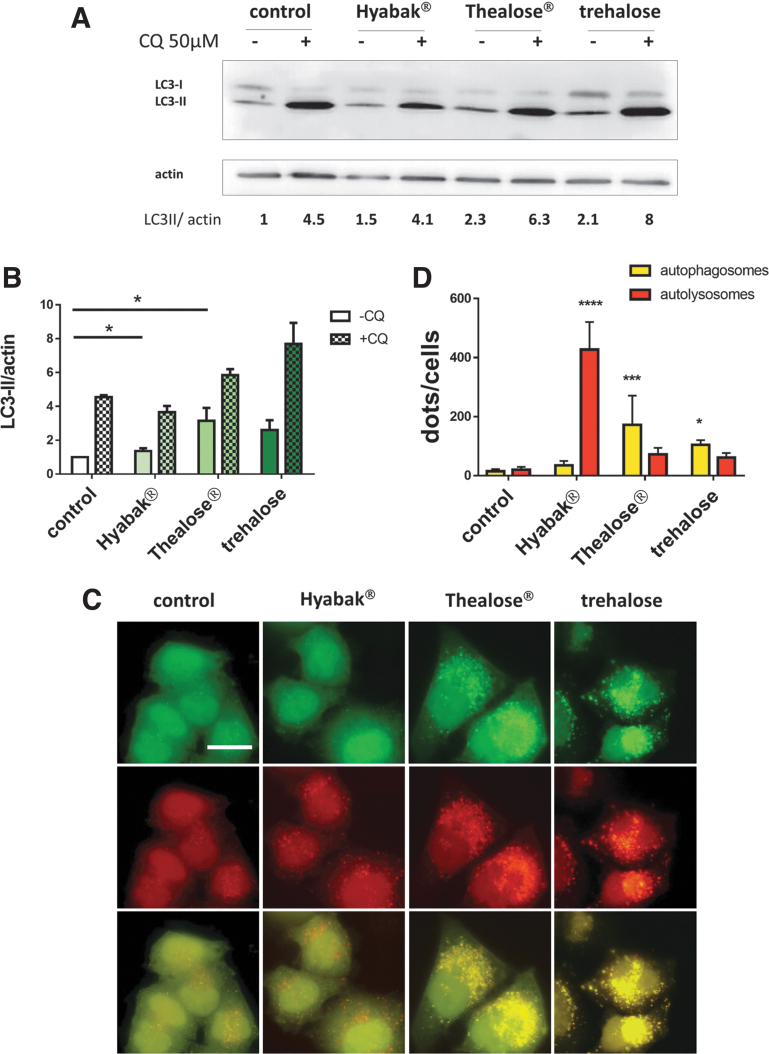
Artificial tears induce the autophagic flux. **(A)** Immunoblot analysis of LC3 expression in HeLa cells, 24 h after treatment with artificial tears. Cells were treated with CQ when indicated to block autophagic degradation. Trehalose was used as a positive control. Actin was used as a loading control. **(B)** LC3-II/actin ratios were quantitated using ImageJ software. Histograms represent the mean ± SD of 3 independent experiments. *P < 0.05 (one-way ANOVA test). **(C, D)** mRFP-GFP-LC3 HeLa cells were treated with artificial tears for 24 h. As a positive control, cells were treated with trehalose. **(C)** Representative images of autophagosomes (*yellow* = RFP+/GPF+ dots) and autolysosomes (*red* = RFP+/GFP− dots). Scale bar = 10 μm. **(D)** Number of autophagosomes (*yellow*) and autolysosomes (*red*) per cell. Histograms represent the mean ± SD of 3 independent experiments. *P < 0.05; ***p < 0.001; ****P < 0.0001 (one-way Anova test). CQ, chloroquine; RFP, red fluorescent protein; SD, standard deviation.

Next, we examined cellular expression levels of LC3 by immunoblot analysis (Fig. 2A, B). It is possible to distinguish the 2 forms of LC3, the nonconjugated form (LC3-I) and the lipidated form of LC3 (LC3-II) based on their electrophoretic mobility.^21^ The relevant parameter in this assay is the ratio between the amount of LC3-II and actin (loading control). We observed an accumulation of LC3-II in cells treated by artificial tears containing SH and trehalose and in cells treated with trehalose alone, confirming our microscopy results.

### Eye drops containing SH with and without trehalose induce the autophagic flux

Autophagy is a dynamic process leading to the degradation of autophagosomal contents into autolysosomes, hence, it is important to verify that accumulation of autophagosomes is indeed correlated with autophagic degradation activity, by measuring the autophagic flux. The autophagic flux can be measured by inferring LC3-II turnover by western blot in the presence and absence of CQ. CQ neutralizes the lysosomal pH and by inhibiting endogenous protein degradation causes the accumulation of LC3-II in autolysosomes. The relevant parameter in this assay is the ratio in the amount of LC3-II in the presence and absence of CQ, which can be used to examine the transit of LC3-II through the autophagic pathway. During autophagic flux, the amount of LC3-II is higher in the presence of CQ. As given in Fig. 2A, treatment by the 2 different artificial tears led to an increased accumulation of LC3-II, suggesting the presence of an autophagic flux.

To confirm our data, the autophagic flux was then investigated using human epithelial cells stably expressing mRFP-GFP-LC3 (Fig. 2C, D). GFP fluorescence is sensitive to acidic pH and is quenched in the acidic environment of autolysosomes, whereas pH has less effect on mRFP. Based on the differing pH stability, this probe allows differentiation of green/red merged images between autophagosomes (GFP+ RFP+ or yellow dots) and autolysosomes (GFP− RFP+ or red dots).^24^ Cells were treated with trehalose alone, and as expected, we observed an increase in the total amount of autophagic vacuoles (autophagosomes and autolysosomes) reflecting an induction of the autophagic flux. Treatments with Thealose Duo or trehalose alone increased the total number of both autophagosomes and autolysosomes. However, addition of trehalose to SH further increased the total number of autophagosomes and autolysosomes. Surprisingly, Hyabak treatment also showed a greater number of autophagic structures and an unusual dramatic accumulation of autolysosomes.

**FIG. 3.**
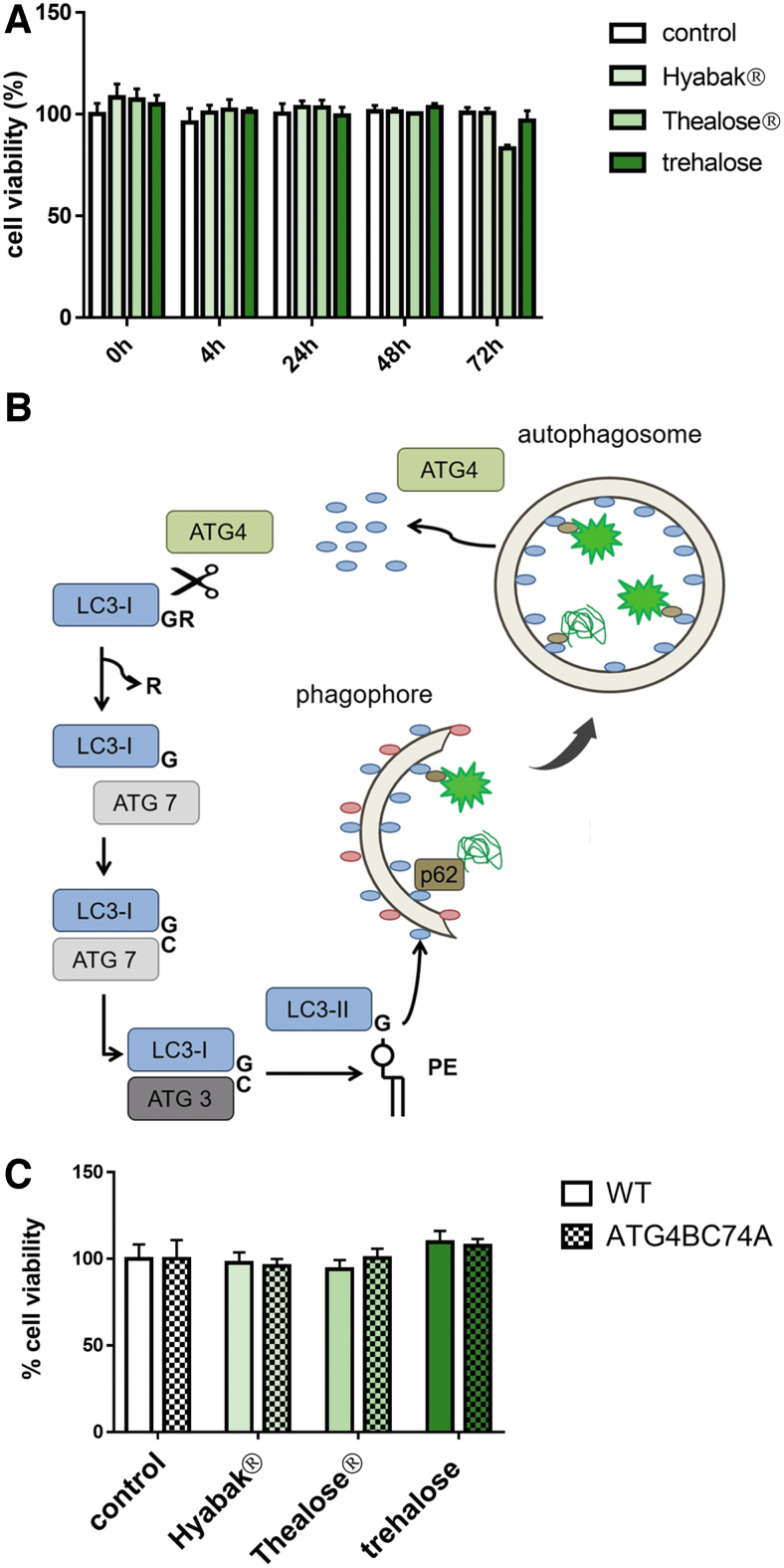
Artificial tears exposure has no impact on autophagy-deficient cell viability. **(A)** Cell viability evaluated by MTT method after exposure of GPF-LC3 HeLa cells at the indicated times. **(B)** Schematic representation of the autophagy process and impact of expression of dominant negative ATG4B (ATG4B C74A). **(C)** Cell viability after overnight artificial tears exposure was evaluated by MTT method. WT or autophagy-deficient (ATG4B C74A) cells. **(A, C)** Histograms represent the mean ± SD of 3 independent experiments. MTT, thiazolyl blue tetrazolium bromide; WT, wild type.

The viability of HeLa cells was assayed in the presence of the 2 different eye drops or with trehalose alone (Fig. 3A). Treatment with Hyabak, Thealose Duo or trehalose did not affect cell viability of HeLa cells compared with control conditions, even after 3 days of exposure. We therefore can conclude that stimulation of autophagy by eye drops does not induce autophagic cell death.

Taken together, our results demonstrate that artificial tears containing SH with or without trehalose stimulate the autophagy process, leading to degradation of autophagic substrates into autolysosomes.

### Autophagy is involved in the protection against desiccation by artificial tears

We investigated whether artificial tears containing SH alone or in combination with trehalose protect cells against desiccation and whether autophagy plays a role in this protection. To avoid any putative interference between the compounds of the artificial tears and an autophagy pharmacological inhibitor, we used a cell line deficient for autophagy. ATG4B is a protease crucial for the processing the lipidation and the recycling of LC3 (Fig. 3B). A mutated form of ATG4B in position C74A impedes its activity and therefore the autophagic process. We thus used a human epithelial cell line stably expressing ATG4B C74A in which autophagy is deficient.^21^ Initially, the viability of wild type (WT) and ATG4B C74A cells was assayed in the presence of the 2 different eye drops or with trehalose alone (Fig. 3B). Treatment with Hyabak, Thealose Duo or trehalose did not affect cell viability of WT cells compared with control conditions (Fig. 3C). Treatment with Hyabak, Thealose Duo or trehalose, had no effect on cell viability of autophagy-deficient cells (ATG4B C74A) in comparison with control conditions (Fig. 3C). In conclusion, exposure to artificial tears does not impact cell viability in normal conditions.

**FIG. 4.**
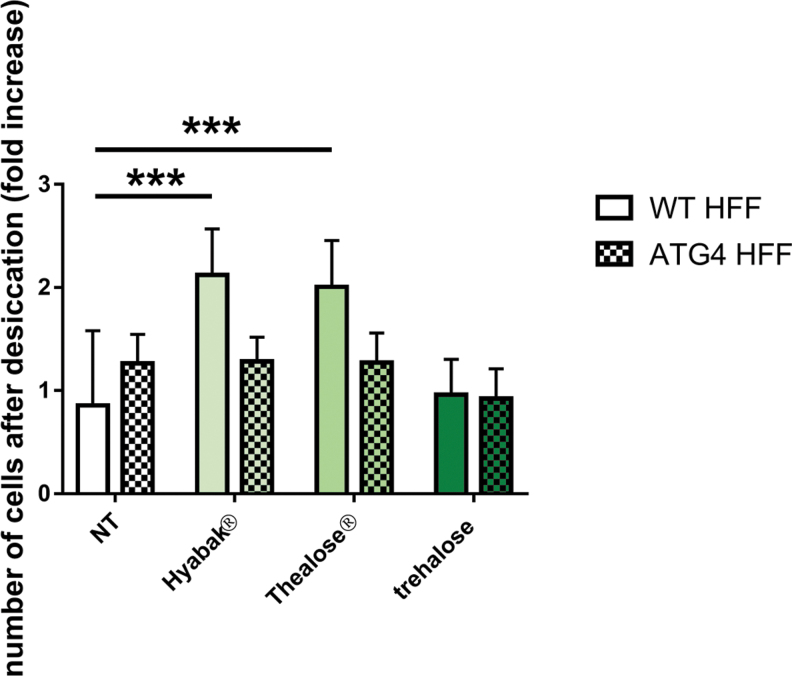
Autophagy is required for protective effect of artificial tears against desiccation. WT and autophagy-deficient (ATG4B C74A) cells were overnight exposed to artificial tears before desiccation. Cell viability was evaluated by MTT method. Histograms represent numbers of surviving cells after desiccation (in fold changes compared with untreated control cells). Results indicate mean ± SD of 3 independent experiments. ***Statistically significant difference compared with the untreated control cells, *P* < 0.001 (one-way ANOVA test). ANOVA, analysis of variance.

To test the protection of artificial tears against desiccation, WT and autophagy-deficient cells were treated with SH or Thealose Duo and then desiccated as described in the Materials and Methods. Figure 4 provides the impact of eye drops treatment on cell viability after desiccation. First, we confirmed that both SH and Thealose Duo treatments allow cell protection against desiccation, whereas trehalose alone has no effect. There were statistically significant increases in the number of cells remaining after desiccation when cells were treated with Hyabak and Thealose Duo compared with the corresponding untreated controls (*P* < 0.001) (Fig. 4). Conversely, in autophagy-deficient cells both SH and Thealose Duo were unable to protect cells against desiccation. These findings demonstrated that artificial tears protect against desiccation in an autophagy-dependent manner.

## Discussion

The etiologic factors for DED include ocular surface, inflammation, tear film instability, and hyperosmolarity.^2^ As hyperosmolarity causes a cycle of inflammation and apoptosis,^25^ it is a potential therapeutic target to improve the patient's symptoms. Hence, osmoprotectants, such as trehalose, likely mitigate corneal epithelial damage and have been incorporated in marketed topical lubricants such as Thealose Duo, which also contains SH. The outcomes of this study indicate that trehalose induces autophagy and the autophagic flux and SH is able to potentiate this induction. In addition, the combination of SH and trehalose reduces the stress caused by cellular desiccation and this protection is dependent on autophagy.

The bioprotectant and osmoprotectant properties of trehalose help maintain homeostasis and counteract the effects of osmotic stress, desiccation, and inflammation.^9^ In response to hyperosmolarity, trehalose participates in balancing the osmotic gradient on either side of cell membranes, limiting water loss.^25,26^ In addition, during desiccation, trehalose allows lipid bilayers stabilization to further reduce water loss and suppress apoptosis.^25^ However, the mechanisms behind the modes of action for trehalose are poorly understood. Trehalose has been shown to be an inducer of autophagy in eukaryotic cells, but is not naturally present in mammalian cells.

We observed that a marketed eye drop (Thealose Duo), containing a mix of SH and trehalose, rapidly induced autophagy in human epithelial cells and that increased in a time-dependent manner out to 24 h. We observed a higher accumulation of autophagosomes within 4 h after incubation of cells with the product, compared with SH or trehalose alone. These observations indicate that SH has a synergistic effect with trehalose for inducing autophagy.

Using CQ as a potent inhibitor of autophagic degradation^27^ we confirmed autophagosome accumulation is owing to the induction of autophagy, and not from an inhibition during the autophagosome maturation process. Hence, trehalose (either alone or in combination with SH) induces an autophagic flux that leads to the degradation of the components contained in the autophagosome vesicles. Of note, SH alone resulted in a large accumulation of autolysosomes indicating a role in autophagy that warrants further investigation.

As the role of autophagy in maintaining homeostasis has a direct impact on cellular survival, we compared the survival of WT and autophagy-deficient cells to desiccation to determine whether it depends on the induction of autophagy. In WT cells, there was a statistically significant increase in the viability of cells treated with SH or SH combined with trehalose, but not with trehalose alone. In autophagy-deficient cells, both marketed eye drops (SH or SH combined with trehalose) lost their protective effect. Taken together, these observations suggest that this protective effect is dependent on the induction of autophagy but treatment with trehalose alone seems insufficient. The pathway involved in trehalose-induced autophagy remains unknown. However, previous studies have reported that trehalose induces autophagy independent of mTOR (mammalian target of rapamycin) and of reactive oxygen species production.^27^ A study of the role of trehalose in human corneal cells has indicated that anti-inflammatory mechanism is independent of the NFκB pathway.^15^

Previous published studies have reported the efficacy of eye drops combining trehalose and SH, both on clinical and biological aspects of DED. Chiambaretta et al.^28^ reported that the combined formulation of SH and trehalose was effective and safe with greater patient satisfaction than SH alone, especially after the first month of treatment. Fariselli et al.^29^ reported a decrease in symptoms of ocular discomfort and corneo-conjunctival surface damage, combined with reduction of tear cytokines levels and recovery of goblet cell density during a 2-month treatment of unpreserved trehalose/hyaluronate tear substitute in DED patients.^29^

Using optical coherence tomography, Schmidl et al.^30^ reported a 6 times longer duration of increased tear film thickness using the trehalose/SH combination, compared with SH alone. In addition, there was longer persistence of the trehalose/SH combination product on the cornea after instillation.^30^ The exploratory prospective evaluation of laser *in situ* keratomileusis patients by Mateo Orobia et al.^31^ indicated that those who received adjuvant treatment with 3% trehalose had statistically significantly better objective and subjective scores for tear film quality than those who received unpreserved SH only.

In addition, we observed that treatment with eye drop containing SH alone was able to both induce the autophagic flux and to protect cells against desiccation in an autophagy-dependent manner. There are currently no data on the effect of SH on the autophagic flux; however, several biological properties of SH have been described *in vivo* and *in vitro*, including promotion of corneal wound healing by initiating corneal epithelial cell proliferation and migration,^32^ and anti-inflammatory properties when SH binds to CD44, a transmembrane glycoprotein.^33^ The relationship between these properties and the induction of autophagy induction requires investigation.

In summary, trehalose combined with SH induces a rapid and sustained autophagic response in epithelial cells and promotes the completion of the autophagic flux.
